# An Emergency Department Quality Improvement Project to Decrease Lumbar Puncture Rates in Febrile Infants 22 to 28 Days Old

**DOI:** 10.1097/pq9.0000000000000749

**Published:** 2024-07-19

**Authors:** Jessica M. Kelly, Brandon C. Ku, Payal Gala, Bobbie Hawkins, Brian Lee, Salvatore Corso, Rebecca Green, Richard Scarfone, Jane M. Lavelle, Emily R. Kane, Laura F. Sartori

**Affiliations:** From the * Division of Emergency Medicine, Children’s Hospital of Philadelphia, Philadelphia, Pa.; † Division of Hospital Medicine, Children’s Hospital of Philadelphia, Philadelphia, Pa.

## Abstract

**Introduction:**

Most providers have routinely performed universal lumbar puncture (LP) on well-appearing, febrile infants 22 to 28 days old. In 2021, the American Academy of Pediatrics recommended clinicians should perform an LP in this age group if inflammatory markers are abnormal. This quality improvement project aimed to decrease LP rates in febrile infants 22 to 28 days old in the emergency department (ED) within 1 year, regardless of race/ethnicity, from a baseline of 87%.

**Methods:**

We used our institution’s quality improvement framework to perform multiple Plan-Do-Study-Act cycles. A multidisciplinary team reviewed the febrile infant literature, local epidemiology, and identified key drivers. We provided departmental education, updated our clinical pathway, and used clinical decision support. We analyzed baseline (January 2017–March 2022) and intervention data (April 2022–March 2024) and tracked data using statistical process control charts. Our primary outcome measure was rates of LP in the ED for this cohort. Process measures included rates of infants with procalcitonin results. ED length of stay, rates of first LP attempt after hospitalization, and missed bacterial meningitis were balancing measures.

**Results:**

The baseline LP rate of 87% decreased to 44% during the intervention period, resulting in a downward centerline shift. There were no significant differences when LP rates were analyzed by race/ethnicity. There was an upward centerline shift in the process measure of infants with procalcitonin results. There was no observed special cause variation in our balancing measures.

**Conclusion:**

Quality improvement efforts, including education, clinical pathway updates, and clinical decision support, safely reduced rates of LPs in febrile infants 22 to 28 days old.

## INTRODUCTION

Accurate identification of febrile young infants (0 to 56 days old) at risk for invasive bacterial infection (IBI), defined as bacteremia or bacterial meningitis, has long been a challenge in pediatric medicine. With physical examinations and clinician judgments being unreliable, more objective risk stratification models have been developed to identify infants at low risk for IBI.^1–4^ Over time, biomarkers such as c-reactive protein (CRP), procalcitonin (PCT), and absolute neutrophil count (ANC) have proven more accurate than white blood cell count in identifying infants at risk for IBI.^2,4–6^ Newer studies show that rates of IBIs decrease with increasing age, with the incidence of IBI in infants in the fourth week of life being 89% lower than in the first week of life.^7^ In addition, due to various factors, including prenatal group B *Streptococcus* screening, *Streptococcus pneumoniae* vaccination, and improved food safety, the epidemiology of bacterial infections in neonates has changed.^6^ In recognition of these advances, the American Academy of Pediatrics (AAP) published a clinical practice guideline (CPG) to help evaluate and manage well-appearing febrile infants. The CPG recommends the use of inflammatory markers (IMs) to risk-stratify infants aged 22 to 28 days in need of further evaluation and treatment for bacterial meningitis, rather than universally performing a lumbar puncture (LP) and administering antibiotics, as was previously standard practice in this age group.^6,8^

The change in recommendations for infants in this age group represents an opportunity to reduce unnecessary medical interventions in a vulnerable population. Beyond lowering the standard procedural risks associated with LPs, including infection, bleeding/epidural hematoma formation, and injury to the spinal cord, the selective use of LPs has the potential to reduce prolonged hospitalizations, exposure to broad-spectrum antibiotics, and associated costs caused by uninterpretable, traumatic, and contaminated cerebral spinal fluid (CSF) results.^6^ Additionally, selective use of LP has an important impact on the patient and family experience, with a reduction in parental anxiety related to invasive procedures in young infants.^9,10^ However, the benefits of not performing an LP must be carefully weighed against the possibility of a delayed diagnosis of bacterial meningitis and increased morbidity.

### Local Problem

Despite the updated AAP CPG recommendations to obtain IMs for risk stratification and LP decision-making, our institution’s providers were not routinely obtaining IMs to guide decision-making for performing LPs. They performed LPs on 87% of febrile infants 22–28 days old. This quality improvement project aimed to decrease LP rates in the emergency department (ED) among febrile infants 22 to 28 days old, regardless of race/ethnicity, within 1 year of project initiation.

## METHODS

### Context

The study institution is a quaternary care children’s hospital system with urban and suburban ED campuses. This project began several months after the opening of the suburban campus and marks the first time that the febrile infant pathway revisions were instituted at both the urban and suburban practice settings.

The urban hospital ED is a large, academic pediatric ED with more than 100,000 visits per year and is a level 1 trauma center. The suburban ED opened in January 2022 with nearly 40,000 visits per year. Both campuses see similar volumes of febrile infants per month. Both EDs are staffed by board-certified pediatric emergency medicine (PEM) attendings who practice at both sites. The urban ED is staffed by pediatric, emergency medicine, and family medicine residents and PEM fellows. Physician assistants, nurse practitioners, and ED pediatricians see patients at both sites. The institution has a robust clinical pathways program with over 190 clinical pathways; 100 are designed for use in the ED. A clinical pathway for evaluating and managing febrile infants has been used since 2010 (https://www.chop.edu/clinical-pathway/febrile-infant-emergent-evaluation-clinical-pathway). In 2014, a revision to the pathway reduced LP rates for infants in the second month of life.^11^ There is associated clinical decision support (CDS) for many clinical pathways, including the febrile infant pathway, within the electronic health record (EHR) in order sets to aid in pathway implementation.

We included patients who were 22 to 28 days old, presenting to either ED with a measured temperature ≥100.4 F at home or during the ED clinical course, and had blood, urine, or CSF cultures obtained during the ED encounter. The 22 to 28 day inclusion was based on the AAP’s separate algorithm for infants 22 to 28 days of age.^6^ We did not exclude patients who were transferred from a referring hospital, were ill-appearing, received recent antibiotics, or had a focal bacterial infection on an exam. We included infants from January 1, 2017–March 31, 2024. We established baseline rates using data from the first five years and began our intervention in March 2022.

### Interventions

A multidisciplinary team collaborated to review the AAP CPG, existing febrile infant literature, and local epidemiology to help identify key drivers. Based on prior work, key drivers identified included clinical pathway updates, associated clinical decision support, and education. Our previous clinical pathway recommended blood, urine, and CSF cultures for all well-appearing febrile infants less than 29 days old. Using our institution’s improvement framework, based on the Model for Improvement framework, we performed multiple iterative Plan-Do-Study-Act (PDSA) cycles to test our change ideas.

Our first intervention focused on education. To educate staff, practice changes were repeatedly presented at division meetings and updates were disseminated via e-mail to those who were unable to attend. Division meetings are attended by physicians, nurse practitioners, physician assistants, and nurses. Both ED sites attend the same faculty meetings, use the same EHR, and have access to the same clinical pathways. The division received the changes well. LP rates were shared at division meetings in October 2022 and April 2023 to provide feedback on this QI initiative. The second educational session focused on cases of febrile infants with differences in management. Providers universally did not perform LPs for normal IMs. However, there was variation in care with subtle elevations in IMs, especially in the setting of a known viral infection or clinical bronchiolitis.

The first PDSA cycle was to update our clinical pathway to align with the AAP recommendations to obtain IMs (PCT, CRP, and ANC) on well-appearing infants 22 to 28 days old. After reviewing the literature, the team defined abnormal IMs at the following levels: procalcitonin >0.5 ng/mL,^2,4^ CRP >2 mg/dL,^2,4^ and ANC < 1000^6^ or >4,000^2^ cells per mm^3^. The cutoff for defining neutropenia was used due to concern for sepsis in young infants with a low ANC.^6^ The updated pathway recommended no LP and hospital admission for observation without empiric antibiotics for infants 22 to 28 days old with normal urinalysis and all three IMs normal. If any IM was abnormal or urinalysis was abnormal, the pathway recommended LP, intravenous antibiotics, and admission. The clinical pathway is available freely online and linked through the EHR^12^. Given prior success using CDS as a higher reliability design concept, the EHR order set was updated with age-based noninterruptive CDS to align directly with the updated clinical pathway and to aid in pathway implementation at the point of care.

We closely tracked IBI and reviewed cases monthly. We reviewed the documented history, physical examination, and laboratory results to ensure safety with initial pathway implementation. There was some confusion for providers in interpretating IM results. The definition of abnormal IM values for febrile infants differed from the hospital's laboratory definition. The EMR is only able to display a single normal reference range for ANC (1600–8290 cells per mm^3^), and flags all values outside of this as abnormal, regardless of the age of the patient. Awareness of this issue was raised with clinicians via discussions in division meetings and via departmental e-mails notices. The authors were identified as key experts in this field. They received frequent responses from clinicians interested in learning more about their care practices and asking for feedback on their practice habits.

After over a year of using three IMs (CRP, ANC, and PCT), we performed another PDSA cycle in August 2023 to recommend using only ANC and PCT. We made this decision after reviewing local data showing that there would have been no missed cases of IBI if CRP was removed, and fewer LPs would have been recommended. In addition, while CRP is included as a recommended IM by the AAP if PCT is unavailable, more recent literature suggests there is insufficient evidence for how best to use CRP.^13^

In response to feedback from division members, we performed a third PDSA cycle to review the available literature regarding bacterial infections in febrile infants with bronchiolitis. The overall low risk of meningitis in the second month of life in infants with clinical bronchiolitis resulted in a change in our recommendations to perform LP in these infants and instead base the decision for LP on individual factors such as clinical appearance and degree of IM elevation. The clinical pathway and order set were updated, and staff were educated on discontinuing CRP use in febrile infants and using individual decision-making based on clinical appearance, number of IMs elevated, and degree of IM elevation for febrile infants with bronchiolitis.

### Measures

Our primary outcome measure was rates of LP attempted or completed in the ED, defined as CSF culture sent from the ED or ED LP procedure note documented (to capture unsuccessful LP attempts). We manually reviewed a year of data and found only one instance where an LP was performed, but no procedure note was documented due to provider error. LP rates were analyzed quarterly given the small monthly number of infants. LP rates were also analyzed by race/ethnicity. Race/ethnicity was determined by extracting demographic data from the EMR. Given small overall numbers, infants were placed in a binary category for demographics (non-Hispanic white versus other race/ethnicity). We graphed LP rates by race/ethnicity to observe overall trends.

Our process measure was rates of infants with PCT results. Balancing measures included ED length of stay (LOS), rates of first LP attempted after hospitalization (defined as CSF culture sent from an inpatient unit), and missed bacterial meningitis (defined as inpatient or return visit LP with growth of pathogenic bacteria in infants who were not empirically treated for bacterial meningitis in the ED). We also evaluated the time to IM result. We used Qlik Sense (Qlik Technologies Inc., King of Prussia, Pa.) for the collection of demographic and laboratory data and RStudio (Boston, Mass.) for the creation of statistical process control (SPC) charts. SPC methodology with standard rules for defining special cause variation was used to assess the effect of these interventions. We used p charts to assess LP rates and PCT results, where the numerator was infants with an LP performed or PCT result, respectively, and the denominator was the total number of infants per quarter. X-bar and S charts were used to assess ED LOS.

We collected data from Qlik Sense and managed it using REDCap electronic data capture tools hosted at the study institution.^14,15^ We reviewed all febrile infant charts in REDCap after the updated pathway was released to evaluate for adverse events (including missed bacterial meningitis, bacteriemia, urinary tract infection, sepsis, and death) using culture data, discharge summaries, and next physician visit >72 hours after the original ED visit.

### Reporting

We used the SQUIRE 2.0 guidelines in the drafting of our article.^16^

### Ethics Approval

This study was exempt from institutional review board oversight as it was a quality improvement project.

## RESULTS

**Fig. 1.**
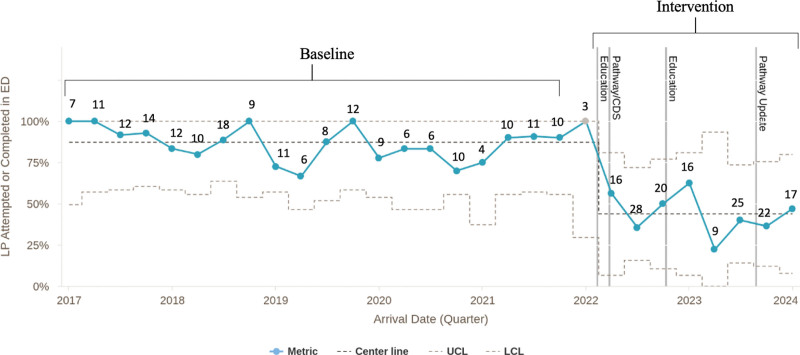
P chart showing rates of LP in the ED by quarter in febrile infants aged 22 to 28 days. The solid blue line indicates rates of LP. Points in gray fall outside of the baseline period and were not included in the calculation of the baseline centerline. The centerline is the dashed dark gray line, and the dashed gray lines show the upper and lower control limits. The gray vertical lines indicate the dates of educational interventions and updated pathway with CDS. The number of patients by quarter is listed in black text near each data point.

**Fig. 2.**
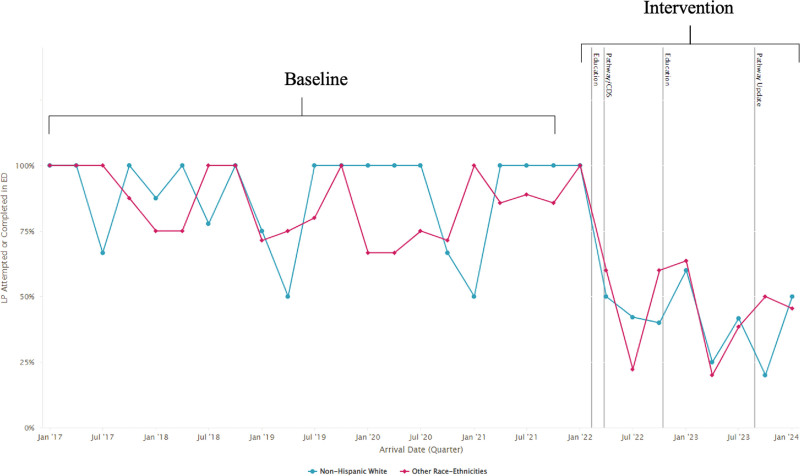
Line chart showing LP rates in the ED by quarter in febrile infants aged 22–28 days stratified by race/ethnicity. The solid blue line indicates rates of LP in patients identified as non-Hispanic White, while the solid pink line indicates LP rates in all other races/ethnicities. The gray vertical lines indicate the dates of educational interventions and updated pathways with CDS. Similar trends in decreased rates of LP were seen regardless of race/ethnicity.

Three hundred fifty-two infants aged 22–28 days were included (Table 1). There were 199 infants in the baseline period and 153 infants in the first 24 months of this quality improvement project. LP rates in infants aged 22–28 days decreased from 87% to 44%, resulting in a centerline shift (Fig. 1). Similar trends in decreased LP rates were seen regardless of race/ethnicity (Fig. 2).

**Table 1.** Patient Characteristics of Febrile Infants 22- to 28-days-Old Evaluated in the ED

	Baseline, N = 199	Revised Clinical Pathway, N = 153
Age, d, median (IQR)	25 (23–27)	25 (24–27)
Evaluated at urban campus, n (%)*	198 (99.5)	82 (53.6)
Non-Hispanic white, n (%)	76 (38.2)	72 (47)
Female, n (%)	77 (38.7)	71 (46.4)
Private insurance, n (%)	98 (49.3)	95 (62.1)
Language other than English, n (%)	8 (4)	9 (5.9)

* Patients were included from two ED campuses, a main tertiary urban campus as well as a suburban campus that opened in January 2022. IQR, interquartile range.

**Fig. 3.**
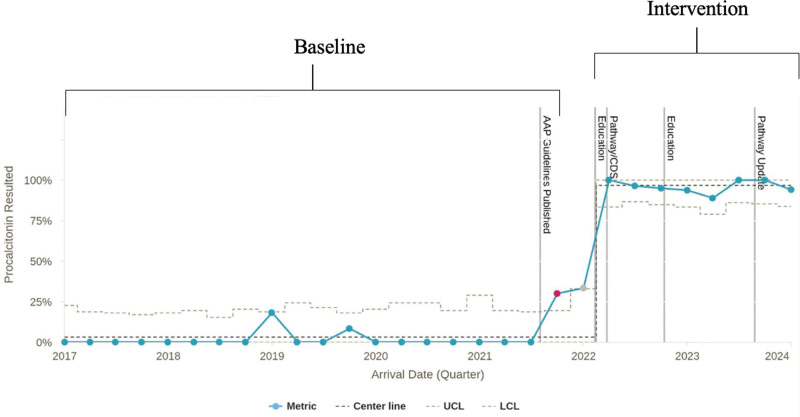
P chart showing the percentage of infants ages 22-28 days old in the ED with a procalcitonin result by quarter. The vertical gray lines indicate the publication dates of the AAP guidelines and our education, pathway, and CDS updates. Points in gray fall outside of the baseline period and were not included in the calculation of the baseline centerline. There was a slight increase in rates of procalcitonin results starting in Quarter 4 2021 (pink dot) after the AAP guidelines were published in Quarter 3 2021; however, a larger and sustained increase was observed with updating the clinical pathway with clinical decision support resulting in a centerline shift.

The baseline rate PCT was 4% (7/199). This increased to 97% (148/153) in our intervention period. Figure 3 is an SPC chart showing the percentage of febrile infants with a PCT result by quarter. After the AAP guidelines were published, there was a slight increase in the percentage of infants with a PCT result; however, with the implementation of this project, a much larger increase was sustained, meeting the criteria for special cause variation and a centerline shift.

**Fig. 4.**
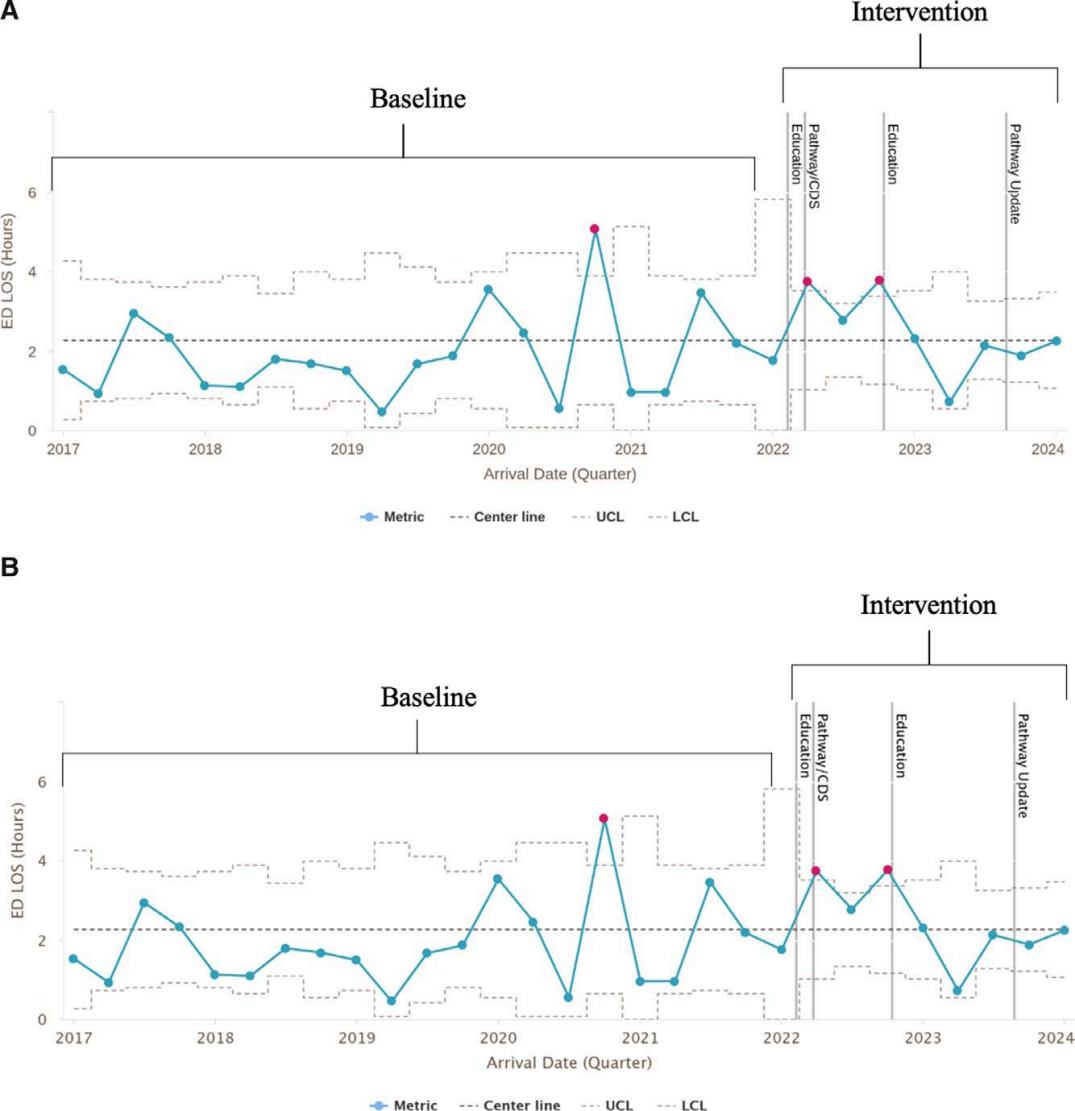
Panel A is an X-bar chart showing average ED length of stay (LOS) in hours by quarter and panel B is an S chart showing the standard deviation of LOS. Solid pink dots indicate points outside the control limits. The centerline is the dashed dark gray line, and the dashed gray lines show the upper and lower control limits. The vertical gray lines indicate the dates of educational interventions and updated pathways with CDS.

Our balancing measure of ED LOS was unchanged with the implementation of this QI project (Fig. 4). Because there was no change in ED LOS despite a reduction in the time-intensive process of performing LP, we evaluated the time to IM result. Across all years, the average time from ED arrival to ANC result was 2.8 hours compared with 3.5 hours for PCT.

There were two patients in the baseline cohort with a first attempt at LP after hospitalization. One infant was in ectopic atrial tachycardia in the ED, and the parent of the second infant declined the LP. After updating the clinical pathway, there were five patients with a first LP attempt after hospitalization; two were unstable from a respiratory standpoint, and two had reassuring IMs but with subsequent changes in clinical course. For these two infants, the inpatient LP results were consistent with viral meningitis. The last infant had normal IMs and was admitted off antimicrobials; however the urine culture was positive (10,000–49,000 CFU/mL Klebsiella pneumoniae). A repeat urine culture and LP were performed inpatient and both cultures had no growth; the initial positive urine culture was felt to be a contaminant per infectious disease. Postintervention, there were no missed cases of bacterial meningitis.

## DISCUSSION

Implementing a local clinical pathway and CDS based on the AAP guidelines reduced LPs in infants 22-28 days old from 87% to 44%, without a significant increase in inpatient LPs or missed bacterial meningitis, effectively and safely reducing unnecessary LPs. Before the publication of the AAP guidelines, previous studies showed high LP rates (>90%) in infants less than a month old, with increasing practice variability and lower rates of LP in older infants.^17^ Our study did not specify a goal for reduction in LP rates, as we were unsure of the baseline rate of abnormal IMs, the primary driver in the decision to LP. This study provides data from a single academic institution on LP rates in the fourth week of life.

Despite the publication of the AAP guidelines, few providers initially used IMs. One reason may be our institutional culture of pathway utilization with order sets, as the order set did not include PCT until our interventions. There is also a known lag in practice change following updates in scientific knowledge.^18,19^ After our interventions, there was a rapid uptake in the use of PCT, resulting in an upward centerline shift at the start of 2022. Our project suggests that using clinical pathways and EHR order sets with noninterruptive, age-specific panels and CDS is an effective way to quickly translate scientific evidence into clinical practice.

Prior literature shows reduced ED LOS by eliminating universal LPs in the second month of life.^11^ In this study, despite a 40% reduction in LPs performed, there was no change in LOS. We surmise the lack of change in LOS was due to not having PCT as rapidly available as ANC, as the turn-around time for PCT was longer. The turn-around time of PCT results can be impacted by practice setting and even time of day; this is an area for improvement, as rapid PCT could decrease ED LOS.^20^

During the chart review, we found that providers had difficulty interpreting IMs. The most common trend noted was incorrect interpretation of ANC, with providers failing to recognize neutropenia (ANC <1000) or subtle elevations of ANC. The challenges of recognizing abnormal IMs highlight the need for easy-to-remember, rounded cutoffs for clinicians.^2^ Also, the discrepancy between laboratory abnormal values for IMs and the pathway-defined abnormal values may make it more difficult for providers. This is an area where the EHR could have specific abnormal values by age and chief complaint to support providers better.

The implementation of our project resulted in a similar number of first LP attempts for inpatients. Patients who required an inpatient LP were either too unstable for the procedure in the ED or had clinical changes necessitating an LP. There was no missed bacterial meningitis when a selective approach to LP was used in this population.

Future research should focus on more opportunities to safely reduce unnecessary interventions in febrile infants while providing equitable, patient-centered care with shared decision-making. For example, the AAP recommends that observation at home be considered for a select group of febrile infants in the fourth week of life. This is a complex calculation of the risk tolerance of both clinicians and families.

Other factors, including implicit bias, factor into the care of infants; one recent study of febrile infants found non-English speaking families were more likely to be admitted to the hospital.^21^ Utilization of clinical pathways and order sets has been shown to decrease disparities in care in adults.^22^ In this study, similar trends in fewer rates of LPs were seen regardless of race/ethnicity. Using a framework similar to the one used is this study may be one approach to equitably care for febrile infants.

### Limitations

There are several limitations to our study. First, despite having data from an urban and suburban ED, the sites are part of a large, academic children’s hospital, limiting generalizability. In addition, our suburban ED opened a few months before this initiative, thus impacting baseline data. Due to the opening of the suburban hospital ED, there was an overall increase in the quarterly volume of febrile infants evaluated at our hospitals. Our institution has a robust clinical pathways program and significant support for such efforts. Other institutions without such a framework may find this implementation strategy difficult due to the time, energy, and resources required.

Although our study did not find any missed cases of bacterial meningitis, it is important to note that our initiative was not powered to detect these differences among the pre- and postimplementation groups. The risk of missed meningitis is not insignificant; delayed treatment of bacterial meningitis results in increased morbidity and mortality.^6,23^ In addition, while the risk of meningitis decreases with age, utilization of a specific age cutoff, such as age greater than 22 days, for a selective approach to LP is arbitrary.^6^ Ultimately, clinicians must carefully weigh the risk of a selective approach to LP in young infants and involve parents in the decision-making regarding the risk–benefit.

Finally, while our first PDSA cycle was to update our clinical pathway for managing well-appearing febrile infants, we defined our cohort as all infants who presented to the ED with fever who had blood, urine, or CSF cultures sent. We included infants the AAP CPG excludes, such as infants with tactile temperature, prematurity, and ill-appearance, because of limitations of our data tracking and the inability to exclude from our data automatically.

### Conclusions

To our knowledge, this is the first study that examined the impact of implementing the AAP’s CPG in the fourth week of life. We safely decreased LP rates, which was sustained over a 2-year period, as our pathway, order sets, and CDS were integrated into provider workflow. Future quality improvement work should focus on other opportunities to bridge gaps in the local care of febrile infants with recommendations from the AAP CPG, such as disposition decisions and antimicrobial therapy.

## ACKNOWLEDGMENTS

*Assistance with the study*: The authors would like to thank the Clinical Pathways Program at Children’s Hospital of Philadelphia for making this work possible.

*Financial support and sponsorship*: none.
